# Double bundle arthroscopic Anterior Cruciate Ligament reconstruction with remnant preserving technique using a hamstring autograft

**DOI:** 10.1186/1758-2555-3-30

**Published:** 2011-12-05

**Authors:** Mitsuo Ochi, Mohamed M Abouheif, Wirat Kongcharoensombat, Atsuo Nakamae, Nobuo Adachi, Masataka Deie

**Affiliations:** 1 Department of Orthopaedic Surgery, Graduate School of Biomedical Sciences, Hiroshima University. 1-2-3 Kasumi, Minami-ku, Hiroshima, 734-8551, Japan; 2 Department of Physical Therapy and Occupational Therapy, Graduate School of Health Sciences, Hiroshima University, 1-2-3 Kasumi, Minami-ku, Hiroshima, 734-8551, Japan

## Abstract

**Background:**

Preservation of the Anterior Cruciate Ligament (ACL) remnant is important from the biological point of view as it enhances revascularization, and preserves the proprioceptive function of the graft construct. Additionally, it may have a useful biomechanical function. Double bundle ACL reconstruction has been shown to better replicate the native ACL anatomy and results in better restoration of the rotational stability than single bundle reconstruction.

**Methods:**

We used the far anteromedial (FAM) portal for creation of the femoral tunnels, with a special technique for its preoperative localization using three dimensional (3D) CT. The central anteromedial (AM) portal was used to make a longitudinal slit in the ACL remnant to allow visualization of the tips of the guide pins during anatomical creation of the tibial tunnels within the native ACL tibial foot print. The use of curved hemostat allow retrieval of the wire loop from the apertures of the femoral tunnels through the longitudinal slit in the ACL remnant thereby, guarding against impingement of the reconstruction graft against the ACL remnant as well as the roof of the intercondylar notch.

**Conclusion:**

Our technique allows for anatomical double bundle reconstruction of the ACL while maximally preserving the ACL remnant without the use of intra-operative image intensifier.

## Background

It is well known that the remnant of the anterior cruciate ligament (ACL) is able to enhance the revascularization and cellular proliferation of the graft, and to preserve the proprioceptive function of the graft construct. Therefore, it seems reasonable to assume that preserving the tibial remnant as much as possible would be a source of reinnervation, and revascularization of the ACL substitute [1,2].

However remnant preservation might be technically difficult, because it might hinder the visualization of the tip of the guide pin used as the first step of the creation of the anatomical tibial tunnel. This is especially true while drilling the posterolateral (PL) tibial bone tunnel because it is located posterior to the anteromedial (AM) bundle insertion. Therefore making a longitudinal slit in ACL remnant would allow better visualization of the tip of the guide pin during anatomical creation of the tibial tunnel [3,4], and avoid the risk of radiation exposure associated with the image intensifier which might be used for anatomical tunnel localization in case of remnant preservation [5,6].

Additionally remnant preservation may run the risk of impingement against the reconstructed graft. Therefore, creating a passage through the remnant using a curved hemostat reaching to the intra- articular aperture of the femoral tunnel, avoids impingement of the graft against the ACL remnant. It was demonstrated that drilling the femoral tunnel through the far anteromedial portal (FAM) leads to more frequent location of the anteromedial femoral tunnel within the anterior cruciate ligament anteromedial bundle anatomic footprint, as opposed to drilling the tunnel trans-tibially. We extended the use of the (FAM) portal for anatomical creation of the both femoral tunnels within the confines of the native ACL femoral footprint. However, we used 3D CT with a special soft ware [Virtual Place Raijin (Aze Ltd., Tokyo, Japan)] for preoperative assessment of the optimal distance of this portal from the medial edge of the patellar tendon. This was verified intra-operatively by inserting a 24 gauge-needle under direct vision from the anterolateral portal into the point predetermined by 3D CT and examining its relation to the cartilage of the medial femoral condyle.

Double bundle ACL reconstruction is increasingly performed nowadays by many surgeons. It has been shown to better replicate the native ACL anatomy and has the biomechanical advantage of better restoration of the rotational stability than single bundle reconstruction. Our developed technique thus allows anatomical double bundle ACL reconstruction with maximal preservation of the ACL remnant, avoids impingement of the reconstructed graft against the ACL remnant, meanwhile maintains the proper angulation of the created tibial tunnel, and avoids radiation exposure associated with the possible use of the image intensifier [7-12].

## Method

### Portal placement

A three portal technique is used, including the standard anteromedial portal, anterolateral portal, and the far anteromedial portal. The anterolateral portal is positioned above the lateral meniscus, adjacent to the lateral border of the patellar tendon. It is utilized as a viewing portal to view the tibial insertion, as well as a working portal. The standard central medial portal is located above the joint line, adjacent to the edge of the inferomedial portion of the patellar tendon. This portal can be used also as a viewing or working portal [4,5,9-13].

**Figure 1:**
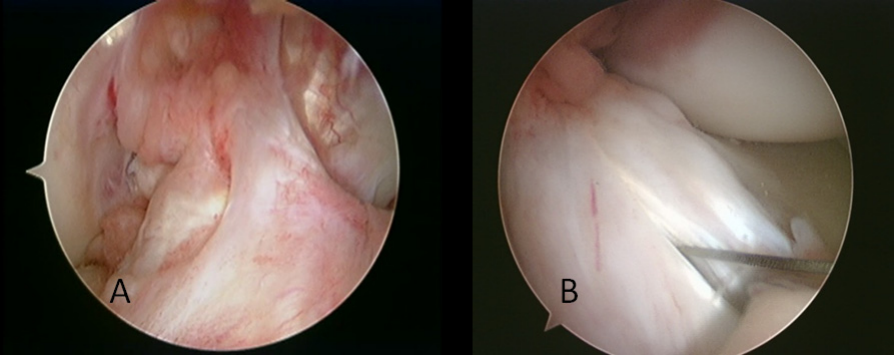
**Arthroscopic view of the ACL remnant**. (A) An ACL remnant bridging between the femur and the tibia viewed through the central AM portal, (B) A longitudinal slit is made in an ACL remnant using an 11 mm blade introduced through the central AM portal while viewing through the AL portal. This would allow visualization of the tips of the guide pins used as the first step of drilling of the tibial tunnels.

For creation of the FAM, 3D CT with a special soft ware [Virtual Place Raijin (Aze Ltd., Tokyo, Japan)] was used for preoperative assessment of the optimal position of the skin incision for this portal that would allow creation of the femoral tunnels within the confines of the anatomical ACL femoral footprint, meanwhile avoid scuffing the articular cartilage of the medial femoral codyle (Nishimori M, et al: Simulated ACL reconstruction with trans-medial portal technique using pre-operative 3D CT -Proposed optimum medial portal position- submitted). Usually the skin incision for this portal is located just superior to the medial joint line about 2-3 cm medial to the medial border of the patellar tendon. The use of this FAM portal for instrumentation allows the central medial portal to be used for viewing the femoral insertion site of both the AM and PL bundle and the lateral wall of the intercondylar notch [5,9-13]. If the anterolateral portal is used as a viewing portal, the central AM portal can be used to make a longitudinal slit in the ACL remnant to accommodate the anatomically created tibial tunnels within the confines of the ACL tibial foot print (Figure 1).

### Arthroscopic Evaluation

We perform routine arthroscopic intra-articular inspections through lateral and medial portals with a 30° oblique arthroscope with the knee flexed at 90° to identify any remaining ACL fibers and determine the nature of their attachment. For the arthroscopic diagnosis of a partial rupture, arthroscopic examination should be performed at various knee flexion angles to consider the different tension patterns of the 2 bundles. The status of the PL bundle femoral insertion can be evaluated with the knee in a figure-of-4 position for good visualization of the femoral attachment of the PL bundle [3,4,14,15].

### Identification of the ACL remnant pattern

The ACL scar pattern was thoroughly examined to determine the treatment strategy and the surgical plan as follows;

› *Group 1;There are no ligamentous continuous fibers in the normal attachment of the ACL to the femur;-*

ACL remnants are either: *Group 1a *The ACL remnant bridging the PCL and tibia. The normal attachment of the ACL to the femur is entirely lost. In *Group 1b; *The ACL remnant bridges the intercondylar notch and tibia. However, in these cases the femoral attachment of the remnant might be attenuated [14].

› *Group 2; Partial ACL rupture with ligamentous continuous fibers in the normal attachment of the ACL to the femur with an ACL remnant that couldn't be ascribed to either the AM or PL bundles (*Figure 1A*)*.

For the cases of both groups, remnant preserving double bundle ACL reconstruction was performed.

### The remnant preserving double bundle ACL reconstruction procedure

#### Femoral tunnel preparation

Through the FAM portal, after cleaning the remaining portion of the anatomic femoral attachment site with a motorized shaver or a curette, a passing pin is directed at the ACL anatomic femoral attachment for the AM and PL bundles. This passing pin is drilled through the femur to emerge on the lateral aspect of the thigh. After over-drilling with the 4.5-mm diameter EndoButton drill, the length of the femoral tunnel is calculated. Then the femoral bone socket is created using a cannulated reamer with the same diameter as that of the proximal portion of the doubled semitendinosus tendon graft. During creation of the femoral tunnels through the FAM, the ACL remnant is carefully retracted medially by a probe introduced through the medial portal.

#### Tibial tunnel creation

The harvest of semitendinosus tendon, as well as the preparation of the site for tibial tunnels is as described before [3,4].

**Figure 2:**
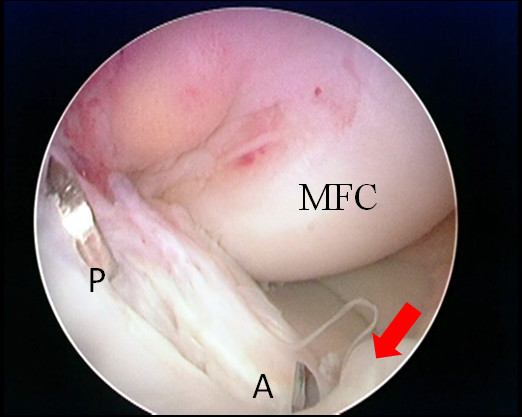
**Drilling of the tibial tunnels through the longitudinal slit in the ACL remnant**. The tip of the guide wire used as the first step for drilling of the PL (P) tibial tunnel appear through the longitudinal slit in the ACL remnant posteriorly, while that for the AM (A) tibial tunnel appear at the anterior end of the slit close to the posterior edge of the transverse intermeniscal ligament (red arrow). Both guide wires are passed through the longitudinal slit in the ACL remnant with the angle of insertion adjusted to be 65° without the need to use intra-operative image intensifier for tibial tunnel localization.

**Figure 3:**
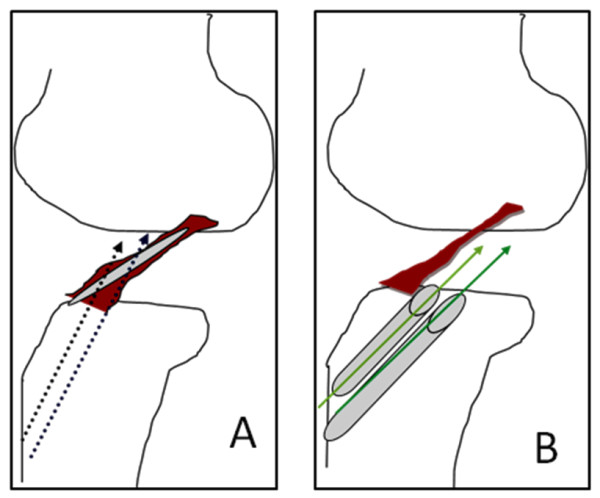
**Importance of longitudinal slit in the ACL remnant and angles of the tibial tunnels**. Schematic drawing showing the importance of making a longitudinal slit in the ACL remnant which together with a more vertical orientation of the tibial tunnels with an angle of 65°. (A) Better visualization of the tip of the guide wires during creation of the AM and PL tibial tunnels through the anatomical ACL tibial footprint. (B) Without the longitudinal slit, and with the ACL tibial guide adjusted to 45°, it would be impossible to visualize the tips of the guide wires used for preliminary drilling of the tibial tunnels as in that case they will be parallel to the preserved ACL remnant.

Two 2.0-mm Kirschner wires are inserted through the anteromedial and the posterolateral portions of the slit made at the tibial attachment of the ACL remnant using the Prot-trac ACL guide system (Acufex, Smith & Nephew, Mansfield, MA) with an angle up to 65° to the tibial plateau to allow visualization of the tip of the wire (Figure 2, 3). If the position of the first Kirschner wire is not appropriate, a second Kirschner wire is added to aim at the desired portion of the tibial anatomic attachment. The transverse intermeniscal ligament is taken as a landmark for placement of the AM tibial tunnel close to its posterior edge. The position of the guide wires is then checked with knee extension [16].

**Figure 4:**
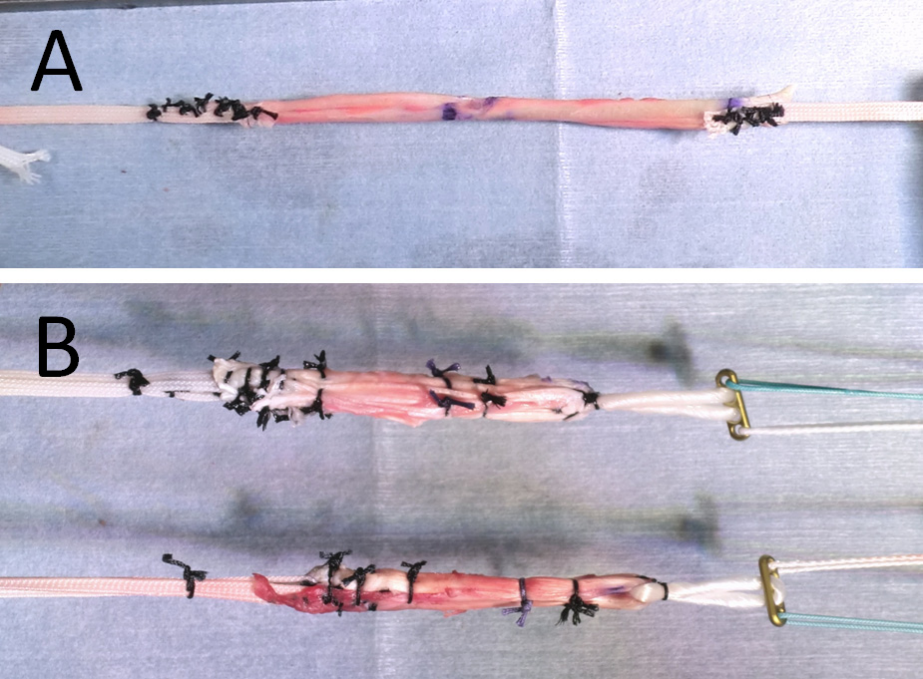
**Preparation of the graft composite**. (A) The semitendinosus tendon was divided in half. EndoButton tapes were mechanically connected in series to each free end of the graft. (B) Each tendon was doubled, and the EndoButton CL was then connected to the loop end.

The appropriate Kirschner wire is then over-drilled using a cannulated reamer of 5-mm or 6-mm diameter to make a tibial tunnel with the same diameter as that of the distal portion of the doubled semitendinosus tendon, which is connected with EndoButton tape (Acufex, Smith & Nephew) (Figure 4). Care should be taken to minimize damage to the ACL remnant by using an arthroscopic probe through the AL portal, while viewing through the AM portal during drilling of the tibial tunnel, and taking care that the tip of the drill pit does not go through the ACL remnant fibers.

**Figure 5:**
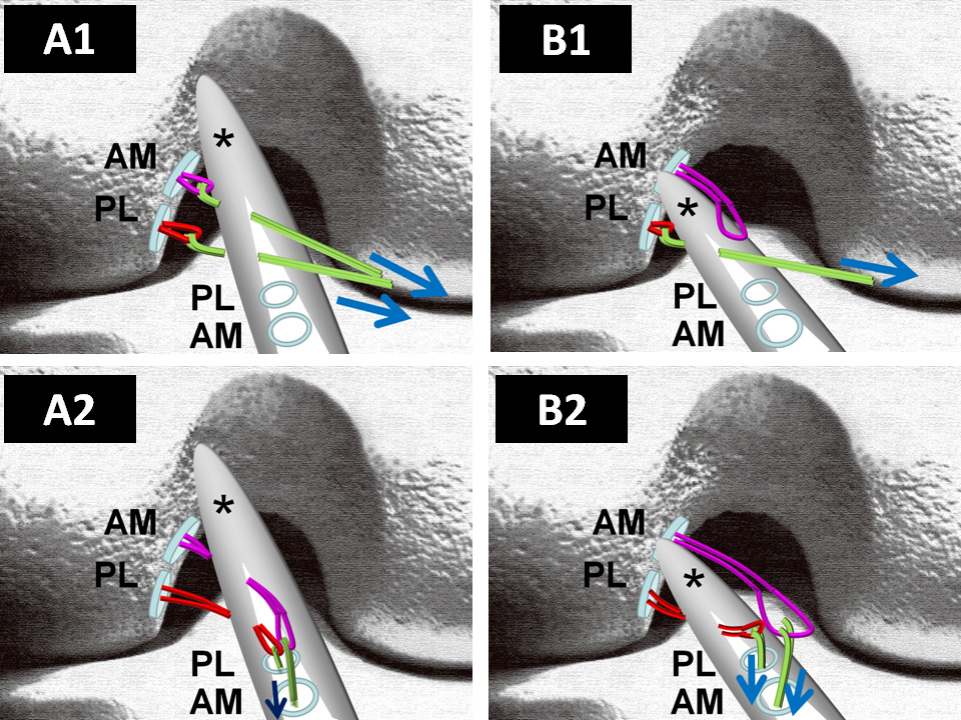
**Creation of the passage through the longitudinal slit made in the ACL remnant**. (A) Schematic drawing showing an ACL remnant (*) attached to the roof of the intercondylar notch (Group 1b). In this case, Ethibond wire loops of both femoral tunnels are retrieved by a curved hemostat through the longitudinal slit in the ACL remnant (A1). An arthroscopic grasper is subsequently used to pass the retrieved wire loops for PL and AM bundle into the created tibial PL and AM tunnels respectively (A2). (B) The ACL remnant (*) is bridging between the tibia and the anatomical femoral footprint (Group 2). In this case, Ethibond wire loop for PL bundle is retrieved by a curved hemostat through the longitudinal slit in the ACL remnant (B1). Subsequently the wire loop for PL bundle is retrieved by an arthroscopic grasper into the tibial bone tunnel for the PL bundle. On the other hand, the wire loop for AM bundle is passed above the ACL remnant, and retrieved by an arthroscopic grasper into the tibial AM bone tunnel (B2). (AM: Bone tunnel for Antero-medial bundle, PL: Bone tunnel for Posterolateral bundle)

Next step is the creation of a passage through the longitudinal slit made in the ACL remnant using a curved hemostat to avoid impingement of the reconstruction graft with the ACL remnants (Figure 5).

**Figure 6:**
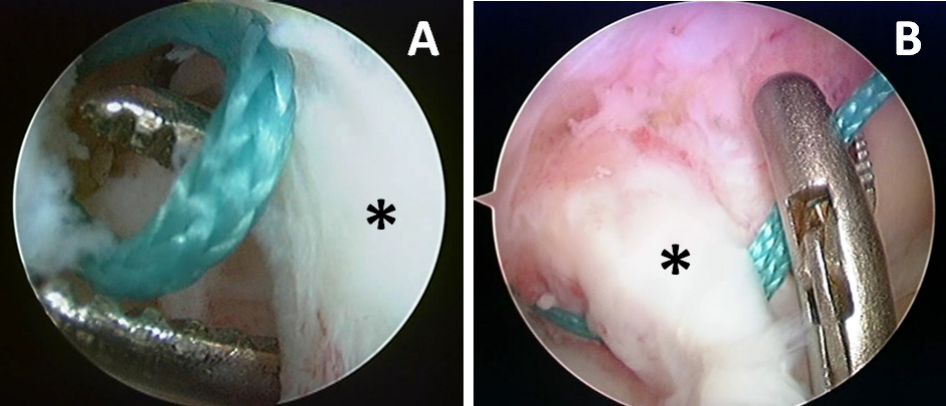
**Passage of the wire loops into the longitudinal slit and tibial tunnels**. (A) Showing retrieval of the wire loop through the ACL remnant (*) using a curved hemostat. (B) This is followed by retrieval of the wire loop by an arthroscopic grasper into the respective tibial tunnel.

If the ACL remnant is attached to the roof of the intercondylar notch or to the PCL (Group 1), a curved hemostat is carefully passed through the slit in the midportion of the ACL remnant from the AM portal to create a passage to the intra-articular aperture of the femoral tunnels. Then Ethibond wire loops are passed into the PL, and AM femoral tunnels respectively through the FAM portal. Subsequently these wire loops are retrieved by a curved hemostat through the longitudinal slit in the ACL remnant (Figure 5A). An arthroscopic grasper is subsequently used to pass the retrieved wire loops into the created tibial PL and AM tunnels respectively. In this case, both graft substitutes are passed through the slit in the ACL remnant (Figure 6).

On the other hand, if the ACL remnant is attached within the anatomical femoral footprint (Group 2), a curved hemostat is carefully passed through the slit in the midportion of the ACL remnant to create a passage to the intra-articular aperture of the PL femoral tunnel, and then an Ethibond wire loop is passed into the femoral tunnel through the FAM portal. Subsequently this wire loop is retrieved by an arthroscopic grasper through the PL tibial tunnel. In this case, the AM graft substitute is passed above the ACL remnant while the PL graft substitute only is passed through the slit in the ACL remnant (Figure 5B, 6).

After selecting an appropriate size of EndoButton-CL (Acufex, Smith & Nephew), the doubled semitendinosus tendon is connected with the EndoButton-CL for the femoral side and EndoButton tape for the tibial side, and the graft composite is completed (Figure 4).

After passing the graft composite through the tibial tunnel and the femoral tunnel, the graft is fixed to the lateral femoral cortex by flipping the EndoButton and pulling the graft distally. Then the knee was fully extended in order to examine impingement of the reconstructed graft or preserved ACL remnant against the intercondylar notch. When impingement was found, the ACL remnant was shaved partially to avoid the possible development of cyclops lesion. After the length change of the graft during knee flexion is examined, the EndoButton tape connected to the graft is fixed to the tibia using two staples (Meira, Nagoya, Japan) with a pulling force of 30 N.

**Figure 7:**
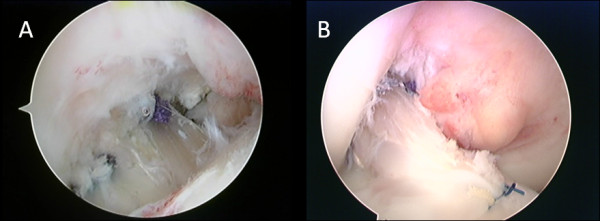
**Arthroscopic view of the double bundle ACL reconstruction with remnant preserving technique**. (A) Showing remnant preserving double bundle ACL reconstruction with both the AM and PL graft substitutes passing through the preserved ACL remnant. B) To optimize this biological reconstruction, the synovium is sutured over the graft-remnant complex to create a closed tube of the reconstructed ACL extending between the tibial and the femoral anatomical foot prints.

**Figure 8:**
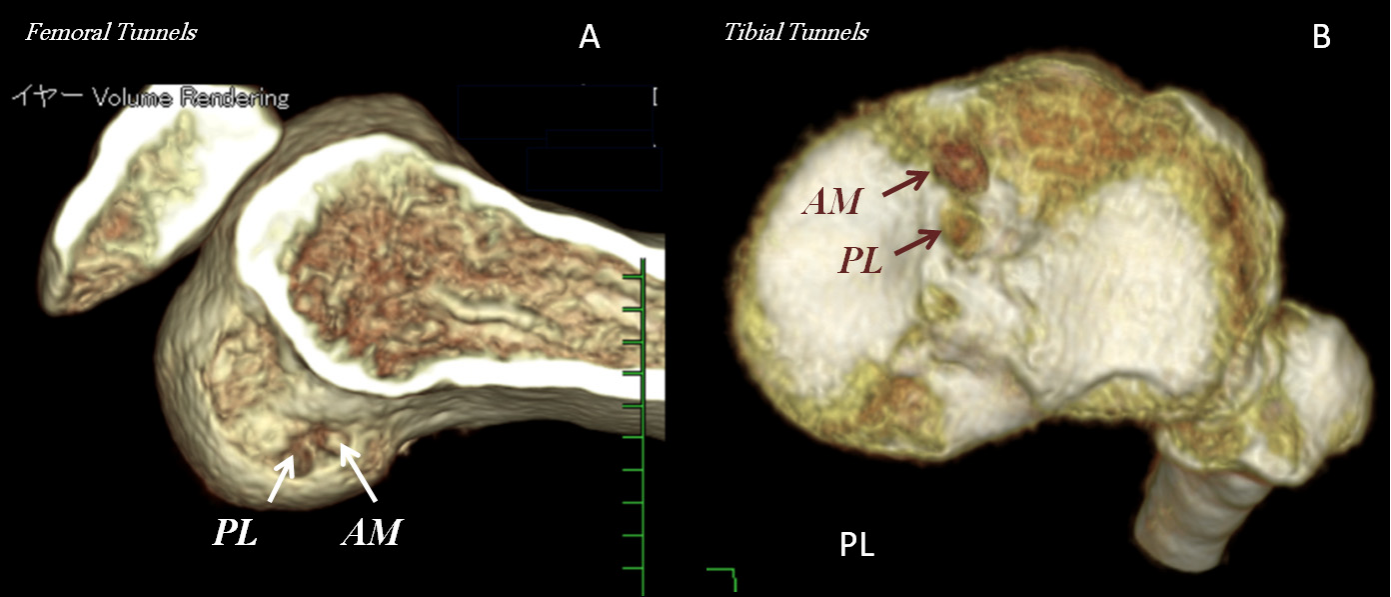
**Post-operative 3D CT images of AM and PL tunnels (A: femoral side, B: tibial side)**. These 3D CT images prove that our technique allows for anatomical double bundle reconstruction without the need for the image intensifier for intra-operative tunnel localization.

Finally, the synovium is sutured over the graft-remnant composite to create a closed tube of the reconstructed ACL extending between the tibial and the femoral anatomical foot prints (Figure 7). Postoperatively 3D CT was used to assess the tunnel position (Figure 8).

## Discussion

In standard ACL reconstruction, even an ACL remnant that bridges the femur and tibia should be debrided to create the femoral and tibial tunnels. However, it has been proven that these remnants conserve the neuroreceptors and mechanoreceptors, which is beneficial to joint position sense after surgery. Adachi et al [1] observed a positive correlation between the number of mechanoreceptors and the accuracy of joint position sense. The authors even found mechanoreceptors in patients having a long interval between the ACL injury and the surgery and concluded that surgeons should consider preserving ACL remnants during ACL reconstruction. Additionally, as far as revascularization of the grafted tendon is concerned, an ACL remnant with abundant vascularity can provide a favorable influence allowing swifter "ligmentization" of the graft [1,2,5,15,17,18].

Crain et al [14] also proved that resection of the ACL remnants, especially those healed to the femur effectively crossing the joint, resulted in a measurable increase in passive anterior laxity in a group of ACL-deficient knees.

Noyes et al [17] reported that in up to 50% of all cases, partial ACL lesions develop into full ligament ruptures, primarily as a result of vascular interruption and necrosis of the fibers following their rupture. Consequently, this type of lesion will sooner or later develop into a full rupture of the ACL, or at the very least force the patient to adopt a lower level of activity.

Augmentation reconstruction is performed in case of partial rupture of the ACL by creating a tunnel that complements the ACL remnant in the native femoral anatomical attachment site. Ochi et al [4] demonstrated the favorable clinical results of the augmentation reconstructive surgery of the ACL using a 1-incision technique. On the other hand, if the ACL remnant is not of considerable thickness, or not bridging the femur and the tibia, remnant preserving double bundle ACL reconstruction can be performed.

Our technique comprises the creation of a longitudinal slit in the ACL remnant fibers. This would allow the visualization of the tip of the guide wire used for preliminary drilling of the tibial tunnel. We also used the transverse intermeniscal ligament as a landmark for creation of the AM tibial tunnel. This would allow for the creation of the tibial tunnels within the native ACL tibial foot print under direct vision without the need to use the image intensifier for tibial tunnel localization. Moreover, reconstruction of the femoral tunnels using the FAM portal independent from the tibial tunnels allow increasing the angulation of the tibial tunnels from 45° to up to 65° to maximize visualization of the tip of the guide wire. Also making a passage using a curved hemostat through the slit in the ACL remnant reaching the intra articular aperture of the femoral tunnel avoids impingement of the reconstruction graft against the ACL remnant and avoids notch overstuffing and the development of cyclops lesion. The biological potential for graft ligamentization is optimized by our technique of suturing the highly vascularized synovial folds over the reconstructed graft-remnant composite [19,20].

During creation of an anatomical tibial PL tunnel with remnant preservation, especially if this remnant is bridging between the femur and the tibia (Group 2), it would be impossible to visualize the tip of the guide pin without making a longitudinal slit in the ACL remnant. The latter would also allow the passage of the PL graft through the remnant with minimal impingement against it. In that case also the AM reconstruction graft can be placed above the ACL remnant. On the other hand, if the ACL remnant is not bridging between the femur and the tibia (Group 1) e.g connecting the tibia to the roof of the intercondylar notch, in that case making a longitudinal slit through the remnant would allow anatomical creation of AM and PL tibial tunnels through the ACL tibial foot print by allowing visualization of the tip of the guide pins used for preliminary drilling of such tunnels, especially if this is coupled with mild increase of the slope of the tibial tunnel from 45 up to 65°.

In this study, the FAM was used to create the femoral tunnels, while viewing through the central anteromedial portal. This resulted in the creation of a femoral tunnel within the anatomic femoral ACL attachment independent of the tibial tunnel. This goes with the findings of other authors who recommended the use of the far anteromedial portal for anatomical creation of the femoral tunnels [4,5,9-13]. However, we added a step of using 3D CT for preoperative prediction of the optimal site of the incision for the FAM portal that allows creation of the femoral tunnels with optimal length and orientation and within the confines of the anatomical ACL footprint meanwhile without damaging the articular cartilage of the medial femoral condyle.

The resident's ridge is arthroscopically identifiable, and is a useful landmark for anatomical femoral tunnel drilling in arthroscopic ACL reconstruction. The main part of the femoral attachment of the ACL is on the resident's ridge, and the remaining part is attached to the posterior portion of the ridge. Creation of the femoral tunnel just on the ridge is theoretically right. However, in ACL reconstruction using hamstring tendons, the center of the femoral tunnel opening is not the central point of application of force, because the graft is pulled to anterior direction. Therefore, we create the femoral bone tunnel just behind the resident's ridge through the far anteromedial portal.

In the future, both short term and long term prospective as well as retrospective clinical studies may be needed to prove the theoretical biological as well as biomechanical advantages of our procedure comprising anatomical double bundle ACL reconstruction, meanwhile with ACL remnant preservation.

## Conclusion

We present a technique that combines the biological advantage of maximal preservation of the ACL remnant and the biomechanical advantage of performing anatomical double bundle ACL reconstruction which offers better rotational stability. This technique was possible through creating the femoral tunnels within the anatomical ACL femoral footprint using the FAM portal. Making a longitudinal slit in the ACL remnant together with increasing the inclination of the tibial tunnels to 65° allow for better visualization of the tips of the guide pins used as the first step of creation of the tibial tunnels within the confines of the anatomical ACL tibial footprint. The creation of a passage through the ACL remnant using a curved hemostat also allows minimal impingement of the reconstruction graft against the ACL remnant, or the roof of the intercondylar notch. Additionally, our technique does not require the use of image intensifier for intra-operative localization of the tunnel position thus avoiding the risk of radiation exposure and making it more convenient for young patients.

## Competing interests

Each author certifies that he has no commercial associations that might pose a conflict of interest in connection with the submitted article.

## Authors' contributions

MO conceived of the surgery, and drafted the manuscript. MMA participated in the surgeries and helped to draft the manuscript. WK, AN, NA and MD participated in the surgeries. All authors read and approved the final manuscript.
